# Mr. Shearly's Case of Peritonæal Inflammation

**Published:** 1806-12

**Authors:** W. Shearly

**Affiliations:** Royal Naval Hospital, Deal


### *To the Editors of the Medical and Physical Journal.*

Gentlemen,

As the following cases appear to me to be curious and important, I shall make no apology for intruding them upon you. It is much to be lamented, that in many singular cases recorded in the Annals of Medicine, of morbid affections attacking the human body, no opportunities presented themselves of tracing symptoms prior to dissolution. As I had, however, the gratification of seeing the subjects of the following cases, at the onset of the disease, I shall relate the symptoms as they occurred during life, and the appearances that presented themselves on dissection. The continual reference of every symptom and suffering (says a popular writer on surgery) to certain physical changes, going on within the body, begets a lively sensibility for the feelings of the patient, while he lives, or to his fate when life is in danger.

### CASE 1.

Joseph Blannan, a seaman, belonging to H. M. gun brig, Pincher, while on duty at the Dock Yard fell down on a log of wood, in which fall his abdomen was struck. Instant vomiting succeeded. It was not done ten minutes before he was brought to the hospital. On examination nothing could be detected externally, as to any marks of violence that had been produced; but, upon placing the hand on the abdomen and making pressure, the most violent and excruciating pain was the consequence. I have just reason to believe he had been drinking a quantity of spirituous liquors, so as to induce intoxication, previous to his meeting with the accident; although at the time I saw him he was perfectly sober: the pain caused by the blow might have produced this. Twenty ounces of blood were abstracted, and his abdomen was ordered to be fomented. An enema was also administered, but no evacuation was produced. An ounce of Castor oil was also prescribed, but this was rejected as soon as taken. He was admitted on Wednesday the 24th of June, 1804, about one o'clock, P. M. The pain continued very severe during the night, and on seeing him the following morning, no mitigation of symptoms had taken place. A warm bath was ordered immediately and an enema to be given as soon as he came out; this brought away a small quantity of hardened fæces. He found himself relieved by the bath, but this was of momentary duration. He had no alleviation from pain from the time he was admitted until the time he expired, but, on the contrary, his sufferings had increased towards the termination of his exisrence. He was perfectly resigned to his fate, and expired about ten o'clock, P. M. on Thursday evening, having lived thirty-three hours from his first receiving the blow.

### DISSECTION.

On opening the abdomen, a quantity of coagulable lymph was thrown out on the surface of the peritonæum which lines the general cavity. This substance was also found between the interstices made by the convolutions of the intestines. The peritonæum was also found in a very inflamed state. The sygmoid flexure of the colon, near to the place where it commences, was found *ruptured*; the opening was about the size of a shilling, through which opening fæculent matter had escaped into the general cavity. On my first detecting this opening, I thought thought I had, through negligence, made an incision into the intestine; but I soon found that I was mistaken, as the aperture was of an oval form, and possessed jagged edges. A serous effusion, to the amount of one pint and a half, was also present. The liver and stomach, as well as the remainder of the abdominal and thoracic viscera, were perfectly healthy.

CASE 2.

William Latham, a seaman, of H. M. S. L'Immortalité, was wounded by a musquet ball on Wednesday night, or early on Thursday morning of the 2d or 3d of October, 1805. He received this wound, while endeavouring to lay a carcase under one of the vessels belonging to the enemy's flotilla. The ball perforated the obliquus externus muscle, about two inches from the superior spinous process of the ileum of the left side, passed through the peritonæum, and lodged under the cuticle near to the great trochanter of the right femur; from which situation it was extracted. It shattered the os innomenatum of the right side in its passage. He was admitted on Saturday, October the 5th, labouring under the following symptoms:

Great tension of the abdomen. The tranverse arch of the colon could be easily seen in the course it took as close as possible to the scrobiculus cordis. He did not complain of much pain on severe pressure being made, but the difficulty of breathing appeared to be the most troublesome symptom that he laboured under. Fæces were discharged from the opening where the ball entered, and a vomiting of bilious matter supervened. Restlessness without fever prevailed, and great anxiety was depicted in his countenance. He rejected all warm liquids and craved for beer, which was given him, and allayed the vomiting considerably. This last symptom did not occur so frequent on the Monday, as it had done the two preceding days, but the fæcal discharge was augmented, and so it continued to his death, which took place on Tuesday October the 8th, about eleven o'clock, A. M. The upper and lower extremities had been cold since Sunday night. Hiccup, and all the symptoms were present that are the result of enteritis terminating in gangrene. Thirty ounces of blood were abstacted a few hours after admission, and contributed towards giving him temporary relief. Fomentations to the abdomen, applied as warm as possible, contributed also to his ease. He voided his urine with great facility, which circumstance appeared, on first sight, to

 be be rather curious, as the direction which the ball took, was directly through the hypogastric region. The bladder therefore, could only have escaped by its being in a collapsed state. This patient's right lower extremity had been paralysed from the first moment he received the shot.

### DISSECTION.

A portion of the intestinum ileum was found wounded, strangulated, and gangrenous, it having protruded itself through the opening which the ball had made in the peritonæum. The portion of intestine was about two inches in length, and could not be returned into the abdomen without dilatation. The large intestines were found distended with air, particularly the transverse arch of the colon, which circumstance easily accounts for its being traced so easy while the man was living. The peritonæal coat of the liver had been evidently inflamed, as small portions, or flocculi of coagulable lymph, were found adhering to its convex surface. The liver and stomach might be said to have been pushed fairly into the cavity of the thorax by the inflated intestines, which easily accounts for the violent dyspnœa with which he was afflicted. I omitted (by its escaping my memory) examining the sciatic nerve, but am fully convinced it was injured, as it appeared almost impossible it should have escaped, from the direction which the ball took; and as paralysis of the lower extremity prevailed, this opinion appears to be well-founded.

### REMARKS.

It must appear obvious in Case the 1st. that the colon must have been distended at the time the blow was received, and that the rupture must be attributed to this distention. Had the blow been severe, surely some external marks of violence would have been discernable; not even the most trifling ecchymosis was present, neither was the structure of the abdominal muscles, situated immediately over the ruptured intestine, at all deranged. The great inflammation which took place was, no doubt, produced by the escape of fæces into the general cavity, and the coagulable lymph found thrown out, was the consequence of such inflammation. This case goes further to prove, how soon this substance is formed, after the first appearances of inflammation take place, and, at the same time, it must convince every practitioner, how active active and decisive the treatment should be in arresting the progress of such inflammatory complaints. Our main chance of success is to check the disease at its onset; and this may be accomplished, *not* by abstracting blood in small quantities, but by taking it from a large orifice in that quantity that shall produce fainting, or that relaxation of the system bordering upon it. There are, however, peculiarities of this disease, in which much must be left to the discretion and ability of the practitioner. The intestine being strangulated in Latham's case, was evidently the reason why his life was prolonged to the extent it was, as the wounded peritonæum, which caused the stricture, retained the intestine in that situation which was productive of an artificial anus, and of course prevented the fæces from escaping into the general cavity. Had this circumstance not taken place, his life must have been curtailed. It is possible the intestine might not have been originally wounded, but that the gut might have become strangulated the moment the patient was shot, and sphacelated prior to his reception. If this was the case, it must have been before his admission, as fæces certainly passed from the opening made by the ball at the time when I first saw him, which was immediately after his reception. Had the portion of intestine shewed itself externally, no time ought to have been lost in dilating the wounded peritonæum, which caused the stricture; and could this have been accomplished soon after strangulation had commenced, the patient might have had some chance of recovery.

I am, &c.

W. SHEARLY.

*Royal Naval Hospital, Deal, Oct.* 23, 1806.

